# Correlation of STAT1 with Apoptosis and Cell-Cycle Markers in Esophageal Squamous Cell Carcinoma

**DOI:** 10.1371/journal.pone.0113928

**Published:** 2014-12-01

**Authors:** Ying Zhang, Yaozhong Zhang, Hailong Yun, Raymond Lai, Min Su

**Affiliations:** 1 Department of Pathology, Shantou University Medical College, Shantou, Guangdong Province, China; 2 Department of Laboratory Medicine and Pathology, University of Alberta, Edmonton, Alberta, Canada; 3 Department of Oncology, University of Alberta, Edmonton, Alberta, Canada; University College London, United Kingdom

## Abstract

We recently found evidence that STAT1 in esophageal squamous carcinoma (ESCC) cells exerts tumor suppressor function, and it regulates five key regulators of apoptosis or cell-cycle progression, including Bcl-2, Bcl-xL, survivin, cyclin D1 and p21. In this study, we confirmed these findings in four ESCC cell lines. Using immunohistochemistry, we also assessed the expression of these proteins in 62 primary tumors. The expression of these markers was heterogeneous, ranging 39 to 69% of the cohort. Significant correlation was found between STAT1 and three proteins (p21, Bcl-xL and survivin), whereas only a trend was identified for cyclin D1 and Bcl-2. We then correlated the expression of these proteins with several clinicopathologic parameters including lymph node metastasis, depth of invasion, clinical stage and overall survival. Significant correlations were found between Bcl-2 and deep invasion (p = 0.033), survivin and lymph node metastasis (p = 0.006), as well as cyclin D1 and clinical stage (p = 0.014). Patients with p21-positive tumors had a significantly longer survival compared to those with p21-negative tumors (p = 0.031). To conclude, our findings support the concept that STAT1 exerts its tumor suppressor effects in ESCC via modulating the expression of key regulators of apoptosis and cell-cycle progression.

## Introduction

Esophageal squamous cell carcinoma (ESCC) is one of the most deadly cancers. This disease is highly prevalent in the Chaoshan area in China, with the annual average, age-standardized incidence rate being 10-folds higher than that of most places worldwide [1]. While it has been suspected that genetic and/or environmental factors may predispose the Chaoshan population to ESCC, the pathogenesis of ESCC remains to be elusive. Recently, we studied the biological signficance of STAT1 in ESCC, since STAT1 has been shown to promote apoptosis and carry tumor suppressor functions in different types of cancers [2]. In support of the concept that STAT1 is a tumor suppressor in ESCC, we found that STAT1 expression is commonly lower in ESCC tumors (67 of 131, 51.1%), as compared to case-matched normal tissue; importantly, a relatively low level of STAT1 expression in ESCC was found to be significantly correlated with a worse clinical outcome, tumor invasion and tumor size [3]. In the same study, gene transfection of *STAT1C* (a constitutively-activated form of STAT1) into two ESCC cell lines (EC1 and EC109), resulted in significant apoptosis, and this biological change correlated with a marked reduction in the expression of several anti-apoptotic proteins and a cell-cycle facilitator (including Bcl-2, Bcl-xL survivin and cyclin D1) as well as an upregulation of p21^Waf1^, a negative regulator of G1 cell-cycle progression [4]. With this background, we hypothesize that STAT1 may mediate its tumor suppressor function in ESCC by modulating the expression of these anti-apoptotic proteins and cell-cycle regulators. While some of these markers have been previously studied in ESCC regarding their clinical significance (e.g. prognosis), their relationship with STAT1 expression in ESCC has not been explored. In this study, we first confirmed the relationship between STAT1 and these five markers in four additional ESCC cell lines. Using immunohistochemistry (IHC), we assessed if the correlation between STAT1 and these markers also hold true in a cohort of patient samples. We also assessed if these markers correlate with various clinicopathologic parameters including the overall survival.

## Materials and Methods

### Patient cohort

This study included 62 consecutive patients with primary ESCC who underwent radical esophageal resection at the Shantou Cancer Hospital from 2003 to 2010. None of the patients received preoperative radiotherapy or chemotherapy. 47/62 (75.8%) were male and 15/62 (24.2%) were female. The median age was 57.8 years (range, 37–75 years). 70-month follow-up data was available for 37 patients; 31/37 (83.8%) died during the follow-up period (median, 29 months). The study was approved by the ethical review committees of the Medical College of Shantou University. All participants involved in our study were given written informed consents.

### ESCC cell lines and culture conditions

Two ESCC cell lines (KYESE150 and KYSE510) and two human esophageal immortalized epithelial cell lines (SHEE and NE3) were included in this study. SHEE were cultured in DMEM supplemented with 10% fetal bovine serum at 37°C under 5% CO_2_, KYSE150 and KYSE510 were cultured in RPMI 1640 and NE3 was cultured in DK-SFM supplement.

### Immunohistochemistry

Envision-Labeled Peroxidase System immunohistostaining was performed as described previously [5]. Briefly, samples were fixed in 10% formalin buffer and embedded in paraffin. Tissue sections (4 µm thick) were steamed in a microwave for antigen retrieval, followed by protein-blocking for 30 min. All slides were first incubated against primary antibody overnight at 4°C, and then treated with secondary antibody for 1 h. Tissues were stained for 3 min with high sensitivity 3,3′-diaminobenzidine tetrahydrochloride, counterstained with hematoxylin, dehydrated and then mounted. The following antibodies were employed: anti-cyclin D1, p21, anti-Bcl-2 and anti-survivin were purchased from Fuzhou Maxim Biotechnology Company (Fuzhou, China). Anti-Bcl-xL (1∶300) was purchased from Cell Signaling Technology, Inc. (Danvers, America).

The staining results were independently evaluated by two pathologists who were blinded to the clinical data. The percentages of positive stained cells were assigned the following scores: 0 (<5% positive cells), 1 (6% to 25% positive cells), 2 (26% to 50% positive cells), 3 (51% to 75% positive cells), or 4 (>75% positive cells). The staining intensity was scored on a scale of 0 to 3 as follows: 0, negative; 1, buff; 2, yellow; and 3, brown. The percentage of positive cells and the staining intensities were then multiplied to generate the immunoreactivity score for each case. Overall staining scores from 0 to 2, 3–6 and ≥7 were considered negative, weak and strong expression, respectively. The weak and strong expressions were considered positive. The expression of cyclin D1 and p21 proteins was considered positive when staining was observed in the cell nucleus. For survivin, Bcl-2 and Bcl-xL, staining in the cytoplasm was considered positive.

### Antibodies and western blotting

Western blot analysis was performed using standard techniques as previously described [6]. The following antibodies were employed: anti-PARP(1∶1000), anti-STAT1 (1∶1000) and anti-p-STAT1(Tyr-701)(1∶1000), anti-FLAG (1∶1000), anti-caspase 3 (1∶1000), anti-survivin (1∶1000), anti- Bcl-2 (1∶1000), anti-p21 (1∶1000) and anti-cyclin D1(1∶1000), all of which were purchased from Cell Signaling (Danvers, MA, USA). Anti-Bcl-xL (1∶1000) and anti-ß-actin (1∶1000) were obtained from Santa Cruz Biotechnology (Santa Cruz, CA, USA). Densitometric analysis was performed using the Image J analysis system (Bethesda, WA, USA); the values for the bands were normalized to those of the β-actin bands.

### Plasmids and cell transfection

FLAG-tagged STAT1C cloned into the backbone of pcDNA3.1 was a gift from Dr. Ouchi (University of New York) [7]. Mammalian expression plasmids for pEF-survivin and Bcl-xL was purchased from Addgene. For each experiment, 1×10^6^ ESCC cells were transiently transfected with 10 µg of *STAT1C*, Bcl-xL or survivin vector or the empty vector (Invitrogen, Burlington, Ontario, CA) in 6-well plates using the lipofectamine 2000 reagent (Invitrogen) as per manufacturer's suggested protocol.

### Cell growth and viability assays

To assess cell growth, ESCC cells were plated at a density of 20,000/ml of culture medium. Cell count, done daily for 4 days, was performed using trypan blue staining (Sigma-Aldrich) according to the manufacturer's protocol. Triplicate experiments were performed independently.

ESCC cells were grown in 96-well plates to 50% confluence followed by plasmid transfection for 48 hours in full media. An empty vector transfection was used as a control. Cell viability was assessed using the MTS Proliferation Assay Kit (Invitrogen) as previously described [6].The absorbance was recorded by a BioRad spectrophotometer at 3 days of cell culture. Triplicate independent MTS experiments were performed.

### Statistical analysis

All statistical analyses were performed using IBM SPSS Statistics 19.0 (SPSS, Chicago, IL, USA). Chi-square and Fisher's tests were used to evaluate the relationship between protein expression and clinical/pathological parameters. Survival was estimated by using Kaplan and Meier curves. The correlation between protein expressions was obtained using a Pearson's chi-squared test. P<0.05 was considered as statistically significant.

## Results

### 1) The relationship between STAT1 and the 5 apoptosis/cell-cycle regulators

**Figure 1:**
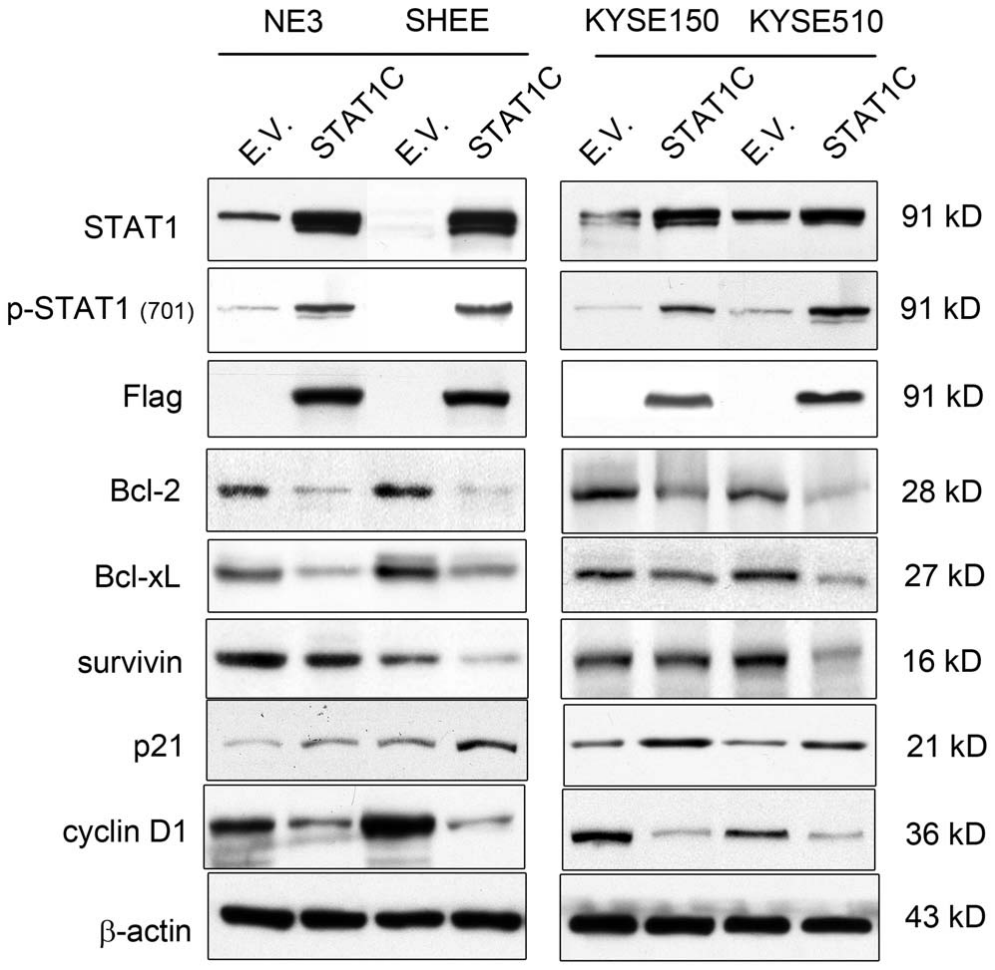
Gene transfection of *STAT1C* upregulated apoptosis in ESCC cell lines. Using Western blot analysis, the gene transfection of *STAT1C* in SHEE, NE3, KYSE150 and KYSE510 cells was shown to be effective, since the levels of STAT1, phospho-STAT1 and FLAG were dramatically increased 2 days after STAT1C transfection. By western blots, gene transfection of STAT1C into these cell lines down-regulated several pro-apoptotic proteins (including BCL-2, BCL-xL, survivin), and promoted G1 cell-cycle arrest by decreasing cyclin D1 and increasing p21waf1. Cell lysates were collected 2 days after the gene transfection of STAT1C in all the cell lines.

As mentioned in the introduction, we observed that enforced expression of the constitutively active form of STAT1 (STAT1C) in two ESCC cell lines substantially modulates five apoptosis/cell-cycle regulators, including Bcl-2, Bcl-xL survivin, cyclin D1 and p21. In this study, we further substantiated this finding by transfecting STAT1C into two additional ESCC cell lines (KYESE150 and KYSE510) and two human immortalized esophageal cell lines (SHEE and NE3). As shown in **Figure 1**, enforced expression of *STAT1C* in these cell lines was confirmed by the high intensity of the total STAT1 band and the strong expression of Flag, which was tagged to the STAT1C construct. Accordingly, there was a marked reduction in the expression levels of Bcl-2, BcL-xL, survivin and cyclin D1, as well as an upregulation of p21. These findings are in parallel with our previous findings using two other ESCC cell lines [3]. Thus, these 5 markers are consistent downstream targets of STAT1 in ESCC cells.

### 2) The biological impact of Bcl-xL and survivin in ESCC cell lines

**Figure 2:**
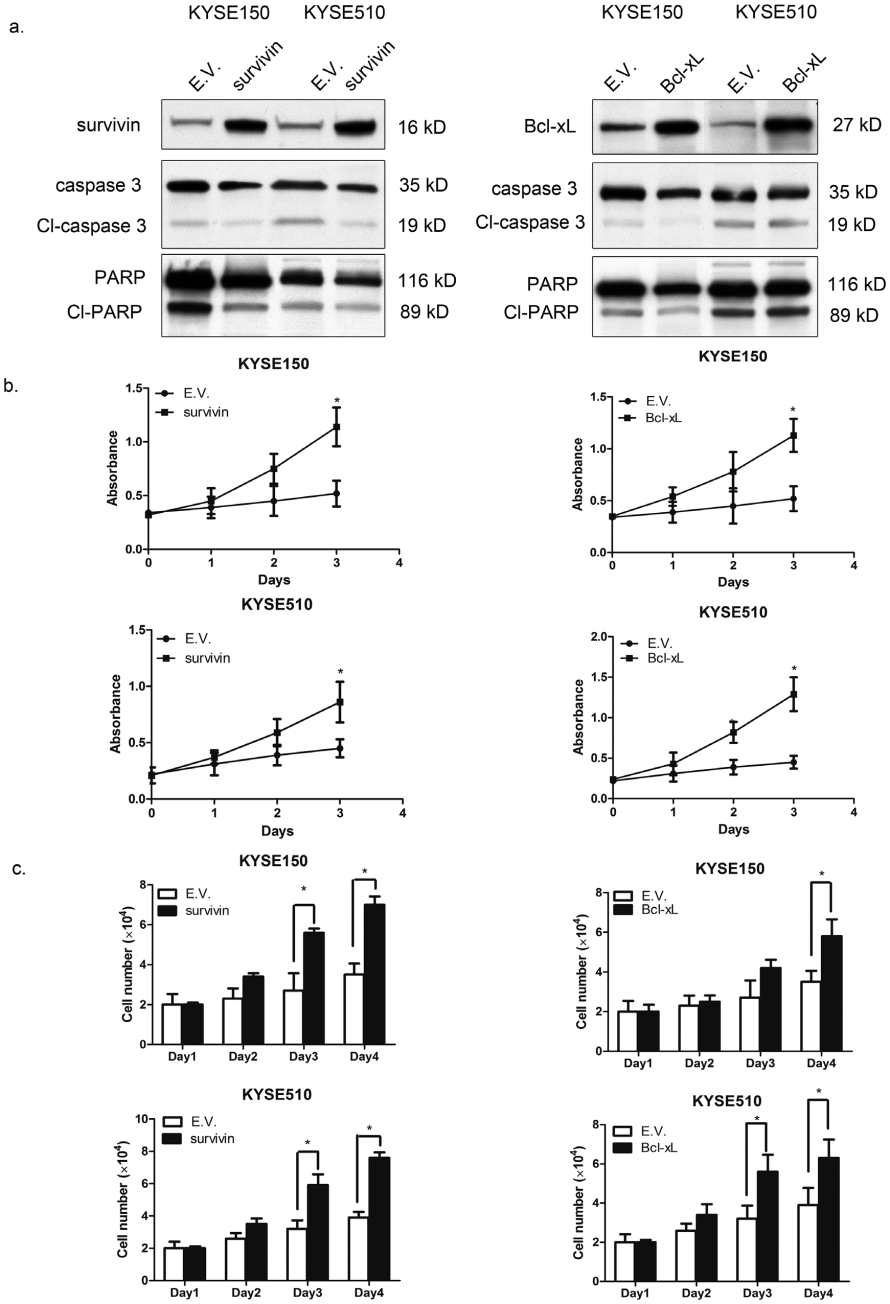
The biology functions of Bcl-xL and survivin in ESCC cell lines. Using Western blot analysis, the gene transfection of *Bcl-xL* and *survivin* in KYSE150 and KYSE510 cells was shown to be effective, since the levels of *Bcl-xL* and *survivin* were dramatically increased 2 days after *Bcl-xL* and *survivin* transfection (a). (b) In both KYSE150 and KYSE510, transfection of *Bcl-xL* and *survivin* induced a significant increase in cell growth, assessed by MTS assay. The cell numbers were assessed on 4 days after transfection. Triplicate experiments were performed independently and the results of a representative experiment are illustrated (* p<0.05). (c) Cell growth, as assessed by trypan blue cell counting, was found to be significantly increased after *Bcl-xL* and *survivin* transfection in KYSE150 and KYSE510 cells (* p<0.05). Triplicate independent experiments were performed.

To support that these STAT1 downstream targets are functionally important, we selected 2 of these 5 targets for functional in-vitro studies. As shown in **Figure 2A**, we transfected Bcl-xL and survivin into KYSE150 and KYSE510 cells. Western blot studies showed that the gene transfection for both vectors was relatively efficient, inducing a relatively high protein level of survivin (left panel) and Bcl-xL (right panel), respectively. Correlating with the induced expression of survivin or Bcl-xL, we found an appreciable reduction in the expression level of apoptotic proteins, including caspase 3 and PARP. We also performed MTS assay, and we found a significant increase in the number of viable cells after Bcl-xL or survivin was transfected into KYSE150 and KYSE510 cells (**Figure 2B**). As shown in **Figure 2C**, using trypan blue to count the number of viable cells, we found that transfection of *Bcl-xL* or *survivin* into KYSE150 and KYSE510 cells led to a significant increase in cell growth, as compared to cells transfected with the empty vector (p<0.05 in both cell lines). These findings have provided support that these STAT1 downstream targets indeed carry biological significance in ESCC.

### 3) Immunohistochemical studies of the 5 markers and STAT1 in ESCC primary tumors

To validate our finding that the 5 markers are indeed downstream targets of STAT1 in ESCC tumors, we performed immunohistochemistry to assess the expression of these proteins and correlated the results with STAT1 expression in a cohort of ESCC primary tumors (n = 62). For Bcl-2, Bcl-xL and survivin, the cytoplasmic immunoreactivity was evaluated and scored. For p21 and cyclin D1, the nuclear staining was evaluated and scored.

**Figure 3:**
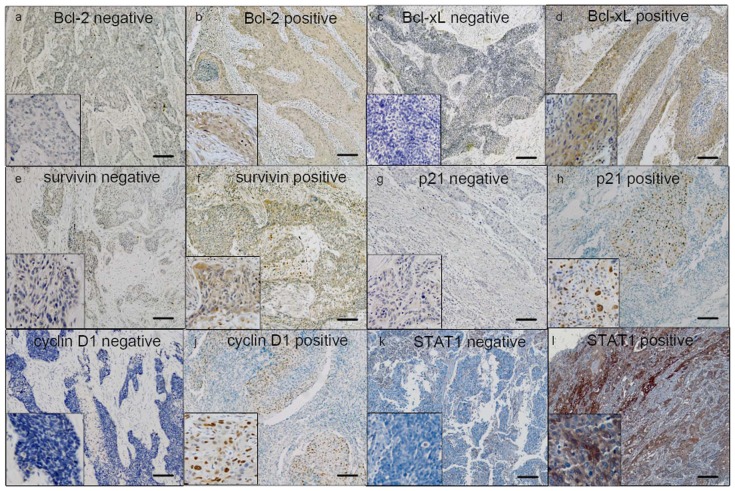
Immunohistochemistry for Bcl-2, Bcl-xL, survivin, p21 and cyclin D1. By immunohistochemistry applied to formalin-fixed paraffin-embedded tissues, all proteins were detectable in most ESCC tumors. The staining was predominantly cytoplasmic for Bcl-2, Bcl-xL and surviving and nuclear for p21 and cyclin D1. Based on the staining intensity, tumors in our cohort were categorized into positive or negative (a) Cytoplasmic negative expression of Bcl-2 (b) Cytoplasmic positive expression of Bcl-2 (c) Cytoplasmic negative expression of Bcl-xL (d) Cytoplasmic positive expression of Bcl-xL (e) Cytoplasmic negative expression of survivin (f) Cytoplasmic positive expression of survivin (g) Nuclear negative staining of p21(h) Nuclear positive staining of p21 (i) Nuclear negative staining of cyclin D1 (j) Nuclear positive staining of cyclin D1(k) Cytoplasmic negative expression of STAT1(l) Cytoplasmic positive expression of STAT1 (IHC stain, scale bar, 20 µm).

Of the 62 cases, Bcl-2, Bcl-xL and survivin were assessed positive in 24 (38.7%), 48 (77.4%), and 35 (56.5%) cases, respectively. For the two cell-cycle regulators, p21 and cyclin D1, positivity was found in 25 (40.3%) and 43 (69.4%) cases, respectively (**Figure 3**). In keeping with our *in-vitro* data, we found a significant inverse correlation between STAT1 and Bcl-xL (r = −0.27; p = 0.036) as well as between STAT1 and survivin (r = −0.29; p = 0.025). Also consistent with our *in vitro* findings, a significant positive correlation was found between STAT1 and p21 expression (r = 0.55; p<0.001). A trend for a positive correlation between STAT1 and Bcl-2 (p = 0.25) or cyclin D1 (p = 0.25) was found (**Table 1**).

**Table 1:** Correlation between Bcl-2, Bcl-xL, survivin, cyclin D1, p21 and STAT1 protein expression in 62 ESCC samples.

	Bcl-2			Bcl-xL			Survivin			p21			Cyclin D1		
Proteins	N	P	r_s_	N	P	r_s_	N	P	r_s_	N	P	r_s_	N	P	r_s_
**STAT1**															
Negative/weak	18	15	−0.148	4	29	−0.267*	10	23	−0.285*	28	5	0.547*	8	25	−0.148
strong	20	9		10	19		17	12		9	20		11	18	

P value for Pearson's X^2^ test; * p<0.05

### 4) The clinical significance of the 5 markers in ESCC

We then assessed whether the expression of Bcl-2, Bcl-xL, survivin, p21 and cyclin D1 correlates with various clinical and pathologic parameters, including gender, location and size of the tumor, lymph node metastasis, histologic grade, depth of tumor invasion/clinical stage and overall survival. As shown in **Table 2**, we found that Bcl-2 expression significantly correlated with the depth of tumor invasion (p = 0.033). The expression of survivin and cyclin D1 significantly correlated with lymph node metastasis and clinical stage (both p<0.05). The expression of Bcl-xL and p21 did not show significant correlation with any of the clinical parameters examined.

**Table 2:** Clinical significance of Bcl-2, Bcl-xl, survivin, p21 and cyclin D1 expression in ESCC.

		Patients		Protein Expression (negative/positive)									
Parameter		N	%	Bcl-2 (38/24)		Bcl-xL (14/48)		Survivin (27/35)		p21 (37/25)		Cyclin D1 (19/43)	
**Age**	≤58	30	48.4	19/11		8/22		13/17		20/10		6/24	
	>59	32	51.6	19/13	0.749	6/26	0.456	14/18	0.974	17/15	0.277	13/19	0.078
**Gender**	Male	47	75.8	27/20		9/38		21/26		28/19		14/33	
	Female	15	24.2	11/4	0.271	5/10	0.253	6/9	0.750	9/6	0.977	5/10	0.795
**Tumor site**	Upper	7	11.3	3/4		2/5		3/4		3/4		0/7	
	Middle	45	72.6	28/17		7/38		18/27		26/19		15/30	
	Lower	10	16.1	7/3	0.512	5/5	0.057	6/4	0.514	8/2	0.272	4/6	0.161
**Differentiation**	Poor	5	8.1	2/3		0/5		2/3		4/1		2/3	
	Moderate	34	54.8	20/14		8/26		13/21		21/13		14/20	
	Well	23	37.1	16/7	0.426	6/17	0.441	12/11	0.573	12/11	0.482	3/20	0.70
**Tumor size**	<5 cm	30	48.4	17/13		6/24		13/17		18/12		6/24	
	≥5 cm	32	51.6	21/11	0.469	8/24	0.638	14/18	0.974	19/13	0.960	13/19	0.078
**Depth of invasion**	T1-T2	14	22.6	12/2		4/10		9/5		9/5		5/9	
	T3-T4	48	77.4	26/22	0.033*	10/38	0.542	18/30	0.075	28/20	0.690	14/34	0.64
**Lymph metastasis**	yes	33	53.2	20/13		8/25		9/24		18/15		6/27	
	no	29	46.8	18/11	0.906	6/23	0.739	18/11	0.006*	19/10	0.380	13/16	0.023*
**Clinical stage**	1–2	28	45.2	18/10		6/22		17/11		19/9		13/15	
	3–4	34	54.8	20/14	0.660	8/26	0.844	10/24	0.013*	18/16	0.233	6/28	0.014*

(*p<0.05).

### 5) The prognostic value of the 5 markers in ESCC

**Figure 4:**
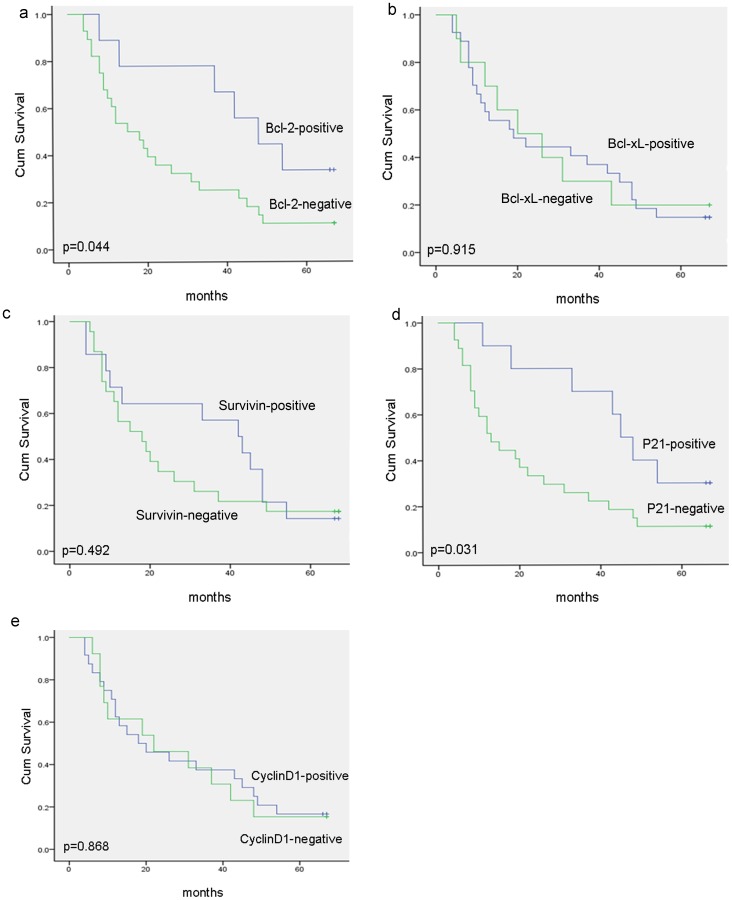
Kaplan-Meier curves of esophageal cancer patients in different positive and negative sub-groups. (a) Bcl-2-positive patients have longer survival than negative groups. (b), (c), and (e) No significant correlation between overall survival and the expression level of Bcl-xL, survivin nor cyclinD1. (d) Significant correlation between overall survival and the expression level of p21 protein levels when the two groups were defined as p21-positive or p21-negative.

Clinical follow-up data was available for 37 of the 62 patients included in this study, with a median follow-up of 29.0 months (range 1–70 months). Survival data was analyzed using the Kaplan-Meier method. The overall survival of patients with Bcl-2-positive tumors (n = 6) was nearly twice as long as those with Bcl-2-negative tumors (n = 28) (44.6 months versus 24.1 months, p = 0.044). In contrast, the survival of patients with Bcl-xL-positive tumors (n = 27) was not significantly different from those with Bcl-xL-negative tumors (n = 10) (29.0 months versus 29.2 months, p = 0.915). Similarly, the survival in the 14 patients with survivin-positive tumors was not significantly different from the 23 cases of survivin-negative tumors (34.7 months versus 25.6 months, p = 0.492). For p21, the overall survival in the positive (n = 7) and negative groups (n = 30) was 45.2 months and 23.1 months, respectively (**Figure 4**), with a significant difference between the two groups (p = 0.031). The median survival rate for patients with cyclin D1-positive tumors (n = 18; 29.0 months) was similar to that for patients with cyclin D1-negative tumors (n = 16, 28.8 months) (p = 0.87).

## Discussion

STAT1, which has been reported to have tumor suppressor functions, is known to regulate cellular differentiation and apoptosis through transcription-dependent as well as transcription-independent mechanisms [8]. Decreased or loss of STAT1 expression has been observed in many types of cancer such as breast cancer, melanoma and leukemia [9]–[11]. One of our recent studies has revealed evidence that the expression of STAT1 is frequently decreased in ESCC, and this abnormality significantly correlates with a worse clinical outcome [3]. Correlating with this clinical observation, our prior *in-vitro* studies using two ESCC cell lines have provided evidence that STAT1 carries tumor suppressor functions in ESCC. Specifically, we found that gene transfection of *STAT1C* into ESCC cell lines effectively induced apoptosis, and this finding was associated with the substantial down-regulation of several pro-survival proteins such as Bcl-2, Bcl-xL and survivin, as well as modulation of two G1 cell-cycle regulatory proteins, p21waf1 and cyclin D1.

In this study, we first validated the correlation between STAT1 and these 5 proteins using 2 additional ESCC cell lines and two immortalized ESCC cell lines. Based on our findings, we concluded that the correlation between STAT1 and these five markers are consistent among all six ESCC cell lines examined (two from our previous study and four from the current study). Importantly, these correlations hold true in patient samples for p21, Bcl-xL and survivin. In addition, a trend was observed between STAT1 and Bcl-2, as well as between STAT1 and cyclin D1, and the statistical significance may have been reached if more cases were included.

Of the five markers, two belong to the Bcl family, namely Bcl-2 and Bcl-xL, both of which are known to play an important role in regulating apoptosis in ESCC [12]. We found that Bcl-2 expression significantly correlated with a long survival in our study. While this finding may appear to be counter-intuitive, we have found another published report describing similar finding in a cohort of ESCC patient samples [12]. However, the correlation of Bcl-2 expression and the survival of ESCC patients is not without controversy. We are aware of two reports describing that Bcl-2 expression in primary-resected ESCC correlates with a worse clinical outcome in these patients [13], [14]. Furthermore, two other reports suggest that Bcl-2 expression is not related to tumor progression nor the prognosis in ESCC [15], [16]. The reason for these discrepancies is likely multi-factorial, but it may be related to the fact that caspases can cleave Bcl-2 into a pro-apoptotic molecule. Thus, in the presence of caspase activation, Bcl-2 is pro-death; in contrast, in the absence of caspase activation, Bcl-2 is pro-survival.

Bcl-xL has been found to be over-expressed in numerous types of cancer, including myelomas, lymphomas, hepatomas, gastric carcinomas and ovarian cancers [17]–[21]. This over-expression is often associated with decreased apoptosis in tumors, resistance to chemotherapeutic drugs and a poor clinical outcome. Down-regulation of Bcl-xL by siRNAs was found to suppress cell growth and induce apoptosis in ESCC cells [22]. Moreover, ESCC patients with high Bcl-xL expression were found to have a significantly shorter survival than those with low Bcl-xL expression [23]. Nonetheless, we found that Bcl-xL expression did not correlate with any of the examined clinical parameters or prognosis in this study. Again, the relatively small number of patient samples included in this analysis may have contributed to the lack of statistical significance. Nonetheless, the possibility of technical difference (e.g. the choice of anti-Bcl-xL and/or antigen retrieval methods) may have accounted for this discrepancy.

Survivin is a member of the inhibitor-of-apoptosis protein (IAP) family and has been reported to promote cell survival and correlate with a worse clinical outcome in different types of cancers, such as breast and colorectal cancer [24]. Several studies suggest a correlation between the expression of surviving and a short survival in ESCC patients [25]. Interestingly, the subcellular location of survivin appears to be important prognostically in ESCC, since nuclear expression was found to have a negative impact whereas cytoplasmic expression has no prognostic relevance [26]. In the present study, we found that survivin correlates with lymph node metastasis and late clinical stage, but has no correlation with the overall survival. The lack of prognostic significance is contradictory with the previous findings that over-expression of survivin in ESCC correlates with poor prognosis [27]–[29]. The reasons for this discrepancy may be due to the small sample size and a relatively short follow up. Further studies with larger sample sizes and longer follow up may help to clarify this issue.

Both cyclin D1 and p21 proteins are involved in regulation of cell cycle progression. Upregulation of cyclin D1 has been shown to shorten the G1 phase and is linked to development and progression of many types of cancer, such as breast cancer, gastric cancer and mantle cell lymphoma [30]–[32]. P21 is a cyclin-dependent kinase (CDK) inhibitor that directly inhibits the activity of the cyclin D1/CDK4 complex. In ESCC, cyclin D1 expression has been shown to be associated with a worse prognosis [33]. However, several studies demonstrate that cyclin D1 is an independent prognostic factor in ESCC, with high cyclin D1 expression having a more favorable prognosis than patients with low cyclin D1 expression [34]. High expression of p21has been shown to correlate with better survival and predicted the response to chemoradiotherapy [35]. The role of p21 as a tumor suppressor is not without controversy. A report has shown that p21 expression correlates with a poor clinical outcome in ESCC patients [36], [37]. Our study demonstrates that expression of cyclin D1 is associated with lymph node metastasis and the clinical stage of ESCC, but p21 nuclear staining has no significant correlation with the survival. Although these results may not be persuasive because of the small sample size, several studies on ESCC also support our findings of a lack of significant correlation between p21 expression and different clinicopathological parameters among ESCC patients [12], [13].

## Conclusions

In summary, the present study primarily evaluated the clinical significance of five proteins, including Bcl-2, Bcl-xL, survivin, p21 and cyclin D1, in a cohort of ESCC patients, by correlating their expression of these proteins with various clinical and pathological parameters. Our overall results support the concept that STAT1 exerts its tumor suppressor effects in ESCC via its modulation of a host of regulators of apoptosis and cell-cycle progression.
